# Recent Advances in Synthesis of Benzothiazole Compounds Related to Green Chemistry

**DOI:** 10.3390/molecules25071675

**Published:** 2020-04-05

**Authors:** Xiang Gao, Jiao Liu, Xin Zuo, Xinyue Feng, Ying Gao

**Affiliations:** 1 College of Geology and Environment, Xi’an University of Science and Technology, Xi’an 710054, China; LJ1379885898@126.com (J.L.); xin_zuo1@163.com (X.Z.); xinyue_feng1@163.com (X.F.); 2 Department of Teaching Quality Evaluation, Yan’an University, Yan’an 716000, China

**Keywords:** benzothiazoles, 2-aminobenzenethiol, green chemistry, condensation, cyclization, thioamide, CO_2_

## Abstract

Benzothiazoles have played an important role in the field of biochemistry and medicinal chemistry due to their highly pharmaceutical and biological activity. The development of synthetic processes is undoubtedly one of the most significant problems facing researchers. In this review paper, we provided recent advances in the synthesis of benzothiazole compounds related to green chemistry from condensation of 2-aminobenzenethiol with aldehydes/ketones/acids/acyl chlorides and the cyclization of thioamide or carbon dioxide (CO_2_) as raw materials, and the future development trend and prospect of the synthesis of benzothiazoles were anticipated.

## 1. Introduction

Benzothiazole is a representative class of sulfur-containing heterocycles and involves a benzene ring fused to a thiazole ring. The benzothiazole ring system was originally found in various marine and terrestrial natural compounds, which is widely used as vulcanization accelerators, antioxidants, plant growth regulators, anti-inflammatory agents, enzyme inhibitors, imaging reagents, fluorescence materials, and electroluminescent devices due to its highly pharmaceutical and biological activity [1,2,3,4]. Especially, benzothiazole plays an important role in the field of medicinal chemistry and renders an extensive range of biological activities including anti-cancer [5,6], anti-bacterial [7,8], anti-tuberculosis [9,10], anti-diabetic [11], anthelmintic [12], anti-tumor [13,14,15], anti-viral [16,17], anti-oxidant [18], anti-inflammatory [19,20], anti-glutamate and anti-parkinsonism [21], anticonvulsant [22], muscle relaxant activities [23], neuroprotective [24], inhibitors of several enzymes and so on [25]. Hence, the synthesis of benzothiazoles is of considerable interest due to their potent and significant biological activities and great pharmaceutical value.

**Scheme 1:**
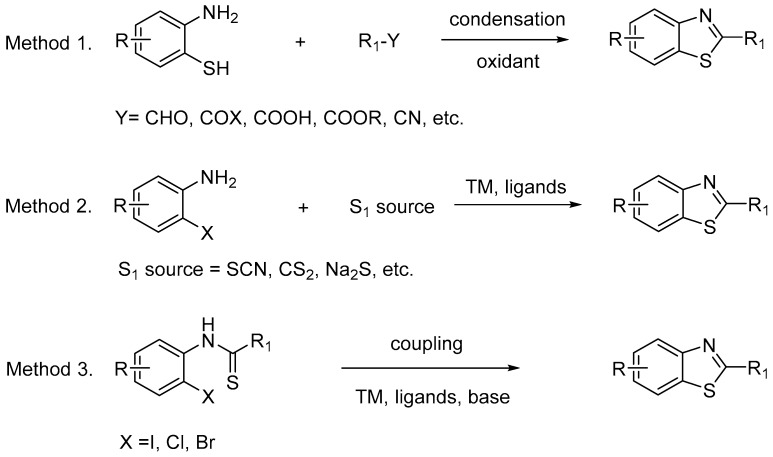
The common synthetic routes for benzothiazoles.

Different synthetic paths have been developed for the preparation of benzothiazole derivatives (Scheme 1). Among them, the condensation reaction of 2-aminobenzenethiol with a carbonyl or cyano group-containing substance is the most commonly used method (Scheme 1, Method 1) [26,27]. Riadi and co-workers [28] found that benzothiazoles could be synthesized from the condensation of 2-aminobenzenethiol and aromatic aldehydes in refluxing toluene at 110 °C. Sun and co-workers [29] reported a copper-catalyzed method for the formation of 2-substituted benzothiazoles via condensation of 2-aminobenzenethiols with nitriles. Moreover, many researchers found that benzothiazoles could also be synthesized by the reaction of ortho-halogenated aniline with isothiocyanates, carbon disulfide and piperidine, aldehydes and sulfur, carbon disulfide and thiol, acid chloride and Lawesson’s reagent (Scheme 1, Method 2) [30,31,32,33,34]. In addition, an alternative method is the intramolecular cyclization of ortho-halogenated analogs (Scheme 1, Method 3) [35,36]. Sahoo and colleagues [37] indicated that benzothiazoles could be synthesized from ortho-halothioureas using both Cu(I) and Pd(II) transition metal as a catalyst. Unfortunately, these traditional processes have some disadvantages, such as low yield, poor selectivity, harsh reaction conditions, the use of toxic reagents or metal catalysts, etc. However, green chemistry advocates for the use of chemical technologies and methods to reduce or stop the use and production of raw materials, catalysts, solvents and reagents, products or by-products that are harmful to human health, community safety, and the ecological environment. Therefore, an exploration of environmentally friendly synthetic routes to prepare benzothiazoles is highly desirable.

In recent years, with the continuous development of green chemistry concepts for the environmental protection [38,39] and resource utilization [40,41,42], the development of metal-free catalysts, the use of renewable reaction materials and reagents, and the realization of reactions under mild conditions have attracted researchers’ attention. Notably, various environmentally friendly pathways have been discovered for the synthesis of benzothiazoles in the past decades. Here, the present article is intended to briefly review recent research progress concerning the synthesis of benzothiazoles compounds, which mainly includes the condensation reaction of 2-aminobenzenethiol and aldehydes/ketones/acids/acyl chlorides. In addition, the cyclization of thioamides offered benzothiazole derivatives. Moreover, a series of benzothiazole compounds were also synthesized by cyclization of CO_2_ as raw materials.

## 2. Synthesis of Benzothiazoles

### 2.1. By Condensation Reaction

#### 2.1.1. Condensation of 2-Aminothiophenol with Aldehydes

**Scheme 2:**
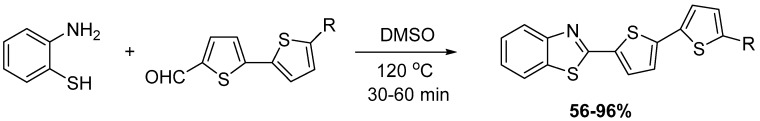
Condensation of 2-aminobenzenethiol with 5-aldehyde bisthiophene compounds.

2-bisthiophene substituted benzothiazole products obtained from the condensation of 2-aminobenzenethiol with 5-aldehyde bisthiophene compounds at the presence of dimethyl sulfoxide (DMSO) under reflux conditions for 1 h was pointed out by Batista and co-authors (Scheme 2) [43]. The evaluation of the fluorescence properties of products was carried out. They have shown strong fluorescence in the 450–600 nm region, as well as high quantum yields and large Stokes’ shifts. Due to their strong fluorescence, these compounds described above could find applications as fluorescent markers.

**Scheme 3:**
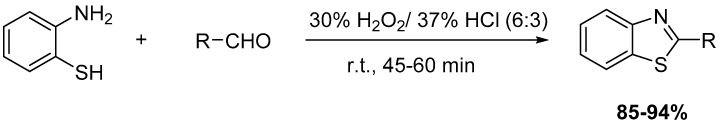
Condensation of 2-aminothiophenol and aldehydes at room temperature.

A series of benzothiazole compounds with different substituents were efficiently synthesized by Guo and co-authors [44] from the condensation of 2-aminothiophenol and aldehydes and their derivatives using a mixture of H_2_O_2_/HCl as a catalyst in ethanol at room temperature for 1 h (Scheme 3). For a comparative study, a ratio of 1:1:6:3 of 2-aminothiophenol/aromatic aldehyde/H_2_O_2_/HCl was found to be optimum for the coupling. Furthermore, both aldehydes bearing electron-donating substituents and electron-withdrawing substituents could be used to obtain the desired benzothiazoles in excellent yields by this method. Short reaction time, easy and quick isolation of the products, and excellent yields are the main advantages of this procedure.

**Scheme 4:**
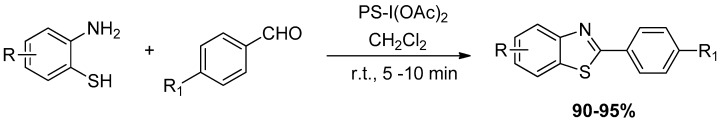
Condensation of 2-aminobenzenethiol and benzaldehyde compounds for 5–10 min.

Kumar et al. [45] found that polystyrene polymer catalysts grafted with iodine acetate could promote the efficient condensation of 2-aminobenzenethiol and benzaldehyde compounds in dichloromethane to synthesize benzothiazole compounds (Scheme 4). The catalyst was synthesized according to an efficient procedure and was used in the combinatorial synthesis of benzimidazoles, which gave the benefits of solid support and the extra advantage of the diversity not being lost in the libraries from any point as in the case of earlier reports. Furthermore, after the reaction, the catalyst was converted to polymer-supported iodobenzene, which was recovered by filtration, converted to poly[4-diacetoxyiodo] styrene (PDAIS) and reused. This reusing could be done many times without loss of activity of the catalyst.

**Scheme 5:**
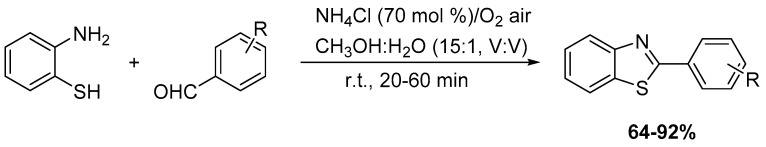
Condensation of 2-aminobenzenethiol and benzaldehydes at room temperature.

Maleki and co-authors [46] found that NH_4_Cl could catalyze the reaction of 2-aminothiophenol with benzaldehyde. Especially, benzothiazole could be obtained at a high yield by this reacting in a methanol-water mixed solvent at room temperature for 1 h (Scheme 5). Furthermore, the study of the mechanism showed that NH_4_Cl activated benzaldehyde through hydrogen bonding and promoted the nucleophilic attack of benzaldehyde by the amino group of 2-aminobenzenethiol to obtain the target product. Obviously, this method is a reaction path that complies with green chemistry for the synthesis of benzothiazole and its derivatives due to short reaction time, high yield, and a recyclable catalyst.

**Scheme 6:**
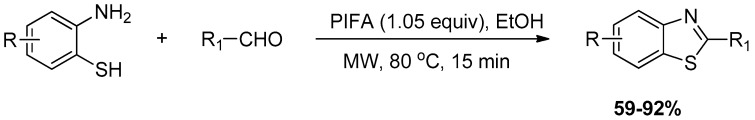
Condensation of 2-aminobenzenethiol and benzaldehydes under microwave irradiation.

Praveen et al. [47] found that the microwave conditions could effectively promote the synthesis of benzothiazole using phenyl iodoniumbis(triuoroacetate) (PIFA) as an oxidation reagent for the condensation reaction of 2-aminobenzenethiol and benzaldehyde compounds (Scheme 6). Compared with the traditional heating method, the usage of a microwave could reduce the reaction time, improve the reaction yield, and expand the universality of the substrate.

**Scheme 7:**
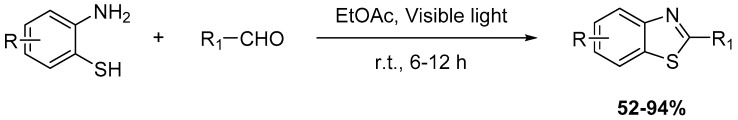
Condensation of 2-aminothiophenols with aldehydes under visible light.

Ye and co-workers [48] have proposed a visible-light-promoted synthesis of benzothiazoles from 2-aminothiophenols and aldehydes (Scheme 7). The reaction system was irradiated with a 12W blue LED for 6 h under an air atmosphere. In order to explore the universality of this reaction condition for different substrates, a series of aldehydes were investigated, and the results showed that aromatic, heteroaromatic, and aliphatic aldehydes are applicable in the transformation. The finding offered an efficient, absence of transition-metal catalysts and extra additive, and a convenient synthetic approach to benzothiazoles.

**Scheme 8:**
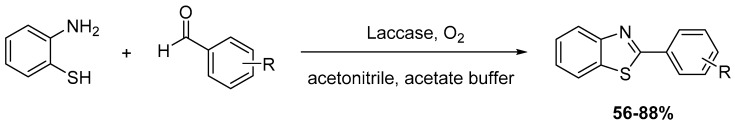
Condensation of 2-aminothiophenol with aryl-aldehydes catalyzed by the commercial laccases.

Maphupha and co-authors [49] have successfully developed a simple and efficient method for the synthesis of 2-arybenzothiazole derivatives in a good to excellent yield from the condensation of 2-aminothiophenol with a series of aryl-aldehydes using an inexpensive commercial laccase as a catalyst at room temperature (Scheme 8).

**Scheme 9:**
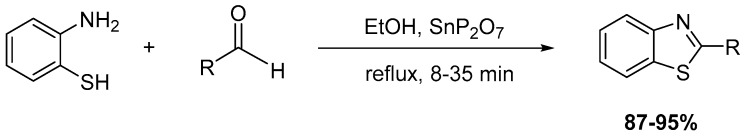
Condensation of 2-aminothiophenol with aromatic aldehydes.

Merroun et al. [50] have reported an efficient, easy, and green method for the benzothiazoles synthesis by a condensation reaction of 2-aminothiophenol with various aromatic aldehydes using SnP_2_O_7_ as a new heterogeneous catalyst (Scheme 9). In this work, the high yields (87–95%) and very short reaction times (8–35 min) were obtained; meanwhile, the new catalyst could be reused at least five times and without any degradation of its activity.

**Scheme 10:**
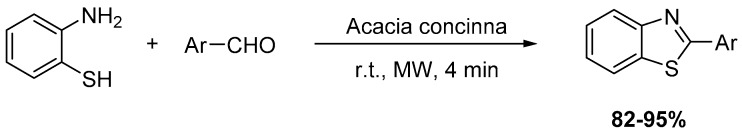
Condensation of 2-aminothiophenol and aryl aldehydes under microwave irradiation.

Bhat and co-authors [51] have investigated the rapid synthesis of 2-aryl-benzothia/(oxa)zoles by the condensation of 2-aminothiophenol and diverse aryl aldehydes in the presence of Acacia concinna as a biocatalyst under microwave irradiation (Scheme 10). In comparison with the conventional method, the microwave irradiation technique has shown a shorter reaction time with higher yields of the anticipated products. Furthermore, the reaction course is quite eco-friendly due to the fact that no solvent is used in this procedure.

#### 2.1.2. Condensation of 2-Aminothiophenol with Ketones

**Scheme 11:**
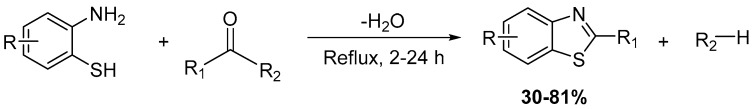
Condensation of o-aminobenzenethiol with ketones.

The condensation of ortho-aminobenzenethiol and its derivatives with representative ketones to yield 2,2-disubstituted benzothiazolines has been investigated by Elderfield and colleagues (Scheme 11) [52]. Notably, pyrolysis of benzothiazoline could yield a 2-substituted benzothiazole and eliminate hydrocarbons under reflux conditions.

**Scheme 12:**
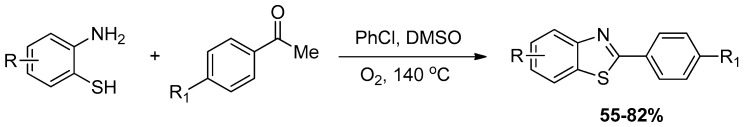
Condensation of 2-aminobenzenethiols and aryl ketones.

Deng et al. [53] have reported an efficient 2-arylbenzothiazole formation from 2-aminobenzenethiols and aryl ketones using molecular oxygen as an oxidant under metal- and I_2_-free conditions (Scheme 12). For a comparative study, the authors have employed various solvents, such as toluene, chlorobenzene, *N*,*N*-dimethylformamide (DMF), DMSO, toluene/DMSO, chlorobenzene/DMSO, and so on. However, chlorobenzene/DMSO has been proven as the best solvent system in terms of yield. Furthermore, this method realized the preparation of benzothiazoles under metal-free and iodine-free conditions and had excellent universality of functional groups, which showed that methyl, methoxy, fluorine, chlorine, bromine, and nitro-substituted raw materials can be successfully converted into a product of the corresponding substituent.

**Scheme 13:**
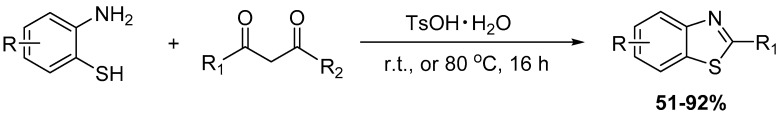
Condensation of 2-aminobenzenethiol with β-diketone.

The effective condensation of 2-aminobenzenethiol with β-diketone was carried out using toluenesulfonic acid as the catalyst under oxidant-, metal-, and radiation-free conditions by Bao and co-authors [54] to offer a series of 2-substituted benzenes in excellent yields (Scheme 13). To screen out the optimal catalytic system, the authors tried different acids, such as benzoic acid (PhCOOH), trifluoroacetic acid (CF_3_COOH), and acetic acid (CH_3_COOH). However, TsOH·H_2_O was chosen as a catalyst for solvent screening in terms of yield. The mechanism showed that the Brønsted acid-catalyzed condensation reaction of 2-amino thiophenol with 2,4-pentanedione would take place to generate a ketamine intermediate in the presence of TsOH·H_2_O. The intramolecular nucleophilic addition and the C−C bond cleavage reaction would finally occur to generate the target product. This method has a host of advantages, including mild reaction conditions, simple experimental steps, simple and readily available raw materials, good substrate universality, and so on, and it has a good application prospect.

**Scheme 14:**
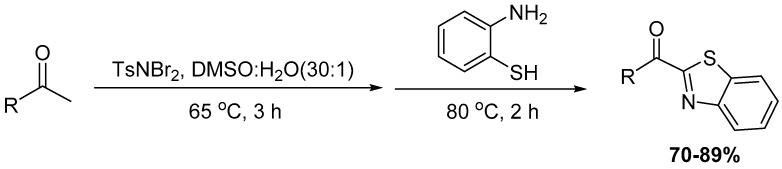
Condensation of aryl methyl ketones and 2-aminobenzenethiol.

An efficient one pot strategy has been developed for the synthesis of 2-acylbenzothiazoles from aryl methyl ketones and 2-aminobenzenethiol under metal-free conditions by Loukrakpam and co-workers (Scheme 14) [55]. The mechanism shows that the aromatic ketones are initially treated with TsNBr_2_ in DMSO at 65°C for 3 h, and the crude reaction mixture is treated with 2-aminobenzenethiol via condensation, Michael addition, and oxidative dehydrogenation sequence to afford the desired products.

#### 2.1.3. Condensation of 2-Aminobenzenethiol with Acids

**Scheme 15:**
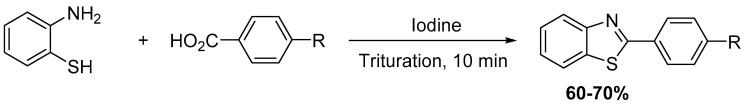
Condensation of 2-aminothiophenol and benzoic acid derivatives.

Gupta and co-workers [56] have reported that molecular iodine was utilized in a one-pot, solid-phase, solvent-free reaction between 2-aminothiophenol and benzoic acid derivatives to obtain an excellent yield of benzothiazole derivatives for 10 min (Scheme 15). Compared with polyphosphoric acid and [pmim]-Br catalyzed microwave synthesis, the new method has a significantly reduced cost due to the fact that no additional chemicals and solvents are essential during the transformation. This methodology has the advantages of being highly economical, less time consuming, and solvent-free.

**Scheme 16:**
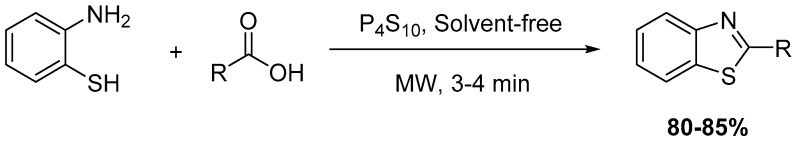
Condensation of ortho-aminothiophenol with fatty acids.

Reuf et al. [57] have reported that the 2-substituted benzothiazoles were prepared by the condensation reaction of ortho-aminothiophenol with various fatty acids under microwave irradiation for 3–4 min (Scheme 16). The reaction was completed with a high yield of the productions using P_4_S_10_ as a catalyst. This protocol is efficient, rapid, and solvent-free.

**Scheme 17:**
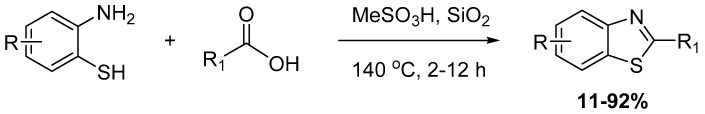
Condensation of 2-aminobenzenethiol with aliphatic or aromatic carboxylic acids.

Sharghi et al. [58] have investigated a high-yielding method for the synthesis of a series of 2-substituted benzothiazole compounds by the condensation of 2-aminobenzenethiol with different kinds of aliphatic or aromatic carboxylic acids (Scheme 17). The novel heterogeneous mixture of methanesulfonic acid and silica gel was developed to be an expeditious medium for the condensation reaction of aromatic and aliphatic carboxylic acids with 2-aminothiophenol for the synthesis of 2-substituted benzothiazoles. In addition, silica could be reused multiple times without reducing the yield. Simplicity, use of widely available and diverse carboxylic acids, and easy handling of the reaction conditions are among the benefits of this method.

**Scheme 18:**
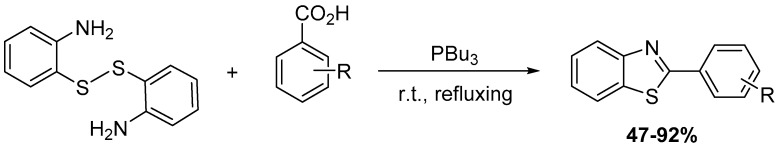
Condensation of 2-aminothiophenol disulfides with carboxylic acids.

Coelho and co-workers [59] have developed a simple and efficient general methodology for benzothiazoles preparation from the condensation of 2-aminothiophenol disulfides with carboxylic acids promoted by tributylphosphine at room temperature (Scheme 18). The universality of this methodology was investigated using 2-aminothiophenol disulfides and various carboxylic acids with donor/withdrawing substituents, which resulted in the desired benzothiazoles with moderate to excellent yields. The advantages of this solution include mild reaction conditions and the addition of non-toxic reagents.

#### 2.1.4. Condensation of 2-Aminobenzenethiol with Acyl Chloride

**Scheme 19:**
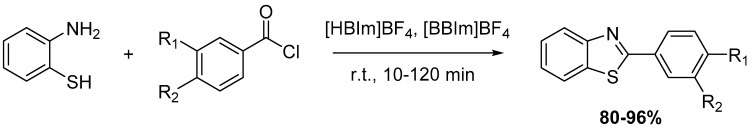
Condensation of 2-aminobenzenethiol with aromatic acid chloride compounds.

Nadaf et al. [60] have suggested an efficient catalyst system, 1-butylimidazole tetrafluoroborate ionic liquid and 1,3-dibutylimidazole tetrafluoroborate ionic liquid for the synthesis of 2-aromatic substituted benzothiazoles through the condensation of 2-aminobenzenethiol with aromatic acid chloride compounds at room temperature (Scheme 19). In this study, several new ILs were synthesized, characterized, and screened for these reactions. The mild reaction conditions, absence of a catalyst, and recyclability of the non-volatile ILs make an environment-friendly methodology amenable for scale up.

**Scheme 20:**
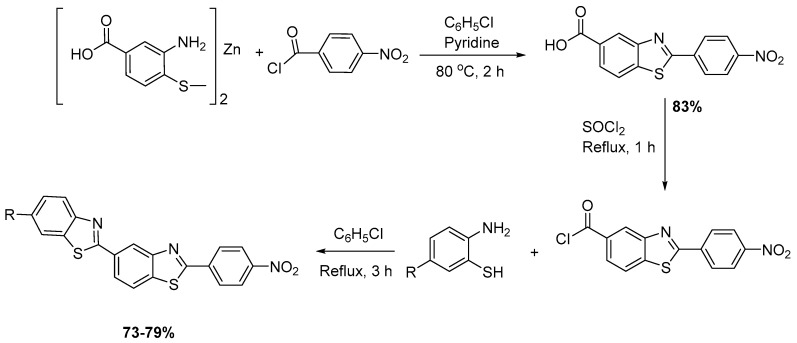
Reaction of 4-amino-3-mercaptobenzoic acid with para-nitro benzoyl chloride.

Wu and colleagues [61] have reported that novel dibenzothiazole derivatives were synthesized. In this work, zinc salt of 4-amino-3-mercaptobenzoic acid was suspended in pyridine with para-nitro benzoyl chloride at 80 °C for an hour and converted into 2-(4′-nitrophenyl)-benzothiazole-6-carbonyl chloride by treatment with thionyl chloride (SOCl_2_) (Scheme 20). Meanwhile, di-benzthiazole-containing compounds were obtained by adding 5-substituted aminothiophenols and heating to reflux for 3 h.

**Scheme 21:**
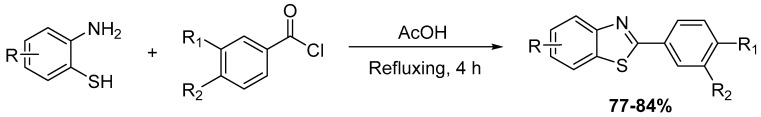
Condensation of nitro-substituted 2-aminobenzothiole with 4-nitrobenzoylchloride.

Racane et al. [62] have reported that the efficient synthesis of nitro-amidino benzothiazoles by a condensation reaction of nitro-substituted 2-aminobenzothiole with commercially available 4-nitrobenzoylchloride under reflux conditions in acetic acid (AcOH) for 4 h (Scheme 21). The target compounds were used as starting compounds for the preparation of targeted amidino substituted 2-phenylbenzothiazole by the Pinner reaction.

**Scheme 22:**
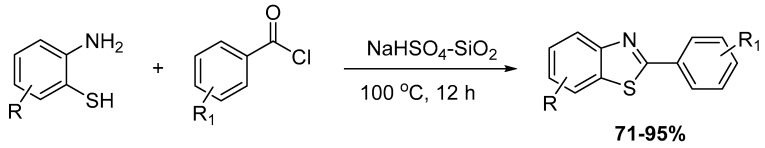
Condensation of acyl chlorides with ortho-aminothiophenol.

An efficient protocol was developed by Kumar and co-workers [63] for the preparation of a library of benzothiazole derivatives from reactions of acyl chlorides with ortho-aminothiophenol in the presence of a catalytic amount of silica-supported sodium hydrogen sulfate (NaHSO_4_-SiO_2_) under solvent-free conditions (Scheme 22). The catalyst NaHSO_4_-SiO_2_ can easily be prepared from the readily available NaHSO_4_ and silica gel (230–400 mesh), and these are inexpensive and non-toxic. Furthermore, as the reaction is heterogeneous in nature, the catalyst could be easily removed by simple filtration. A simple workup procedure, high yield, easy availability, reusability, and the use of eco-friendly catalysts are some of the advantages of the present suggestion.

**Scheme 23:**
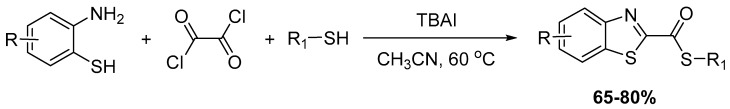
Reaction of thiols, oxalyl chloride, and 2-aminothiophenol.

Dar and co-workers [64] have investigated a synthesis of benzothiazoles using a one-pot three-component reaction between thiols, oxalyl chloride, and 2-aminothiophenol in the presence of n-tetrabutylammonium iodide (TBAI) at 60 °C (Scheme 23). The present protocol favored the formation of the desired products via simultaneous formation of C−N and C−S bonds in good yields with a wide range of substrates. The advantages of this method include mild reaction conditions and high efficiency.

**Scheme 24:**
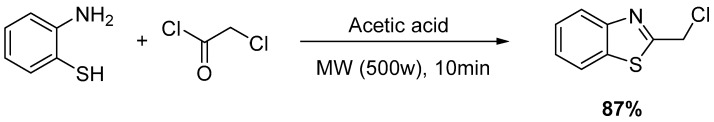
Condensation of 2-aminothiophenols with chloroacetyl chloride.

Luo and co-authors [65] found that 2-chloromethyl-benzothiazole could be obtained from the condensation of 2-aminothiophenols with chloroacetyl chloride in acetic acid under microwave irradiation for 10 min (Scheme 24). Compared with the traditional methods, the microwave-assisted procedures were efficient and environmentally friendly, with less time and high yield.

### 2.2. By Cyclization

#### 2.2.1. Cyclization of Various Substituted Thioamides

**Scheme 25:**
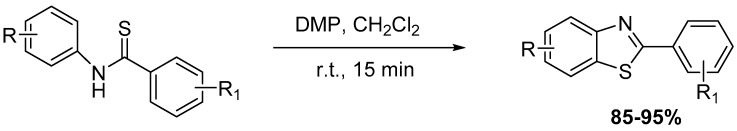
Cyclization of sulfamide substrates.

Bose and co-workers [66] have developed an environmentally beneficial, efficient, and rapid procedure for the synthesis of benzothiazoles via the cyclization of sulfamide substrates in the existence of Dess-Martin periodinane as the catalyst in dichloromethane as the reaction solvent at room temperature for 15 min (Scheme 25). The mechanism shows that the reaction proceeds via a thiyl radical in high yields to give the novel compound oxybis benzothiazole and is also amenable to generating combinatorial libraries of heterocyclic compounds by solid-phase synthesis. This method has the advantages of short reaction cycle, simple operation, good yield, mild conditions, etc.

**Scheme 26:**
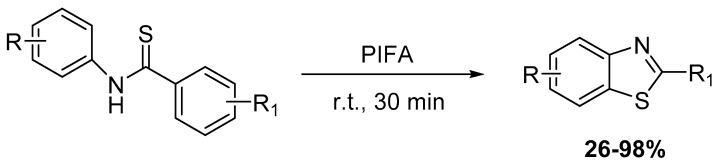
Cyclization of thiobenzamides at room temperature.

Downer and colleagues [67] have investigated a general method for the intramolecular cyclization of thiobenzamides to benzothiazoles via aryl radical cations as reactive intermediates under mild conditions (Scheme 26). In this method, the usage of phenyliodine(III) bis(trifluoroacetate) (PIFA) in trifluoroethanol or cerium ammonium nitrate (CAN) in aqueous acetonitrile was to promote cyclization within 30 min at room temperature in moderate yields.

**Scheme 27:**
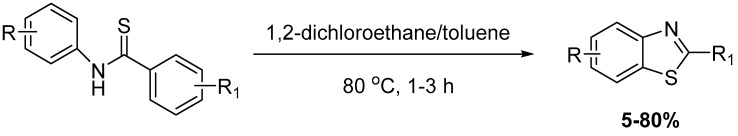
Cyclization of thioformanilides.

Rey and co-workers [68] reported that 2-substituted benzothiazoles were efficiently synthesized by radical cyclization of thioformanilides induced by chloranil under irradiation in 1,2-dichloroethane and toluene at 80 °C (Scheme 27). Especially, hydrogen atom abstraction from thiobenzamide by triplet chloranil was the key step of the mechanism. The protocol was simple, and the heterocycles were easily isolated from the reaction mixture.

**Scheme 28:**
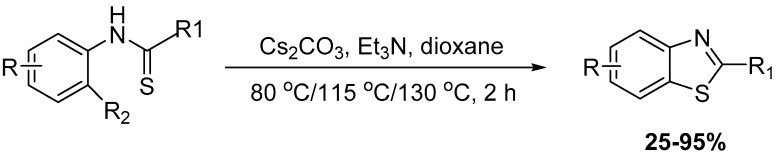
Cyclization of substituted thioformanilides.

An efficient, economical, and convenient method was developed for the preparation of 2-substituted benzothiazoles from *N*′-substituted-*N*-(2-halophenyl)thioureas, *O*′-substituted-*N*-(2-halophenyl) carbamothioates, or *N*-(2-halophenyl) thioamides through a base-promoted intramolecular C–S bond coupling cyclization in dioxane without any transition metal by Feng and colleagues [69] (Scheme 28). A variety of functional groups were tolerated under these conditions and good yields were achieved. This method has some advantages of transition-metal-free, mild reactive conditions, wide application scope, and shorter reaction times.

**Scheme 29:**
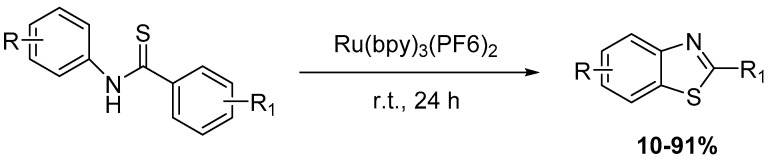
Cyclization of thioanilides.

Cheng and co-authors [70] have developed an aerobic visible-light photoredox synthesis of 2-substituted benzothiazoles by radical cyclization of thioanilides (Scheme 29). The range and functional group compatibility of the new visible-light photocatalytic reaction were studied, and the results showed that the method could tolerate many functional groups and the reaction was very mild. This method has the highlights of unique selectivity, high efficiency, and an environmentally friendly nature.

**Scheme 30:**
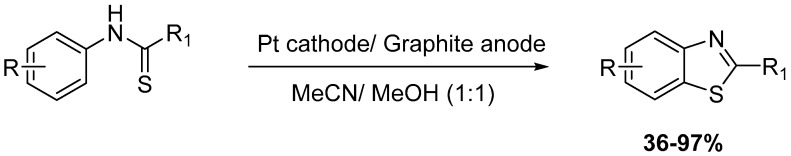
Cyclization of arylthioamides.

Folgueiras et al. [71] have reported that the catalyst-free and supporting electrolyte-free electrochemical synthesis of benzothiazoles has good to excellent yields and with high current efficiencies from arylthioamides using a flow electrochemical reactor (Scheme 30). In this method, an easy scale-up of the reaction without the need for a larger reactor largely improved the reported methods for the formation of benzothiazoles.

**Scheme 31:**
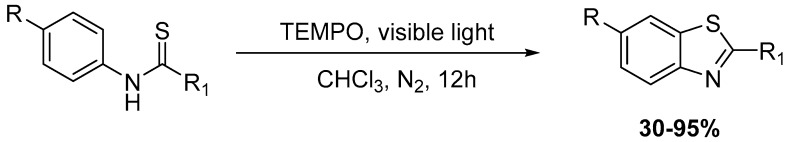
Cyclization of thioamide derivatives.

Xu and colleagues [72] have developed a methodology for visible light-driven, intramolecular C−S bond formation of aromatic substrates to give benzothiazoles through the cyclization of thioamide derivatives in the presence of 2,2,6,6-tetramethylpiperidine *N*-oxide (TEMPO) and obtained excellent yields (Scheme 31). Notably, this photochemical cyclization does not require an extra photoredox catalyst, transition-metal catalyst, or base.

**Scheme 32:**
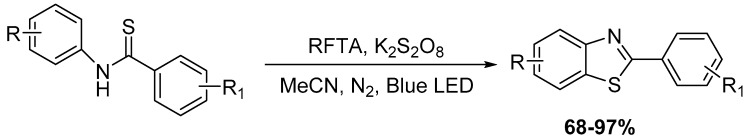
Cyclization of thiobenzanilides under visible light.

Bouchet and co-workers [73] have reported an efficient methodology for the synthesis of benzothiazoles in good to excellent yields from the cyclization of thiobenzanilides using riboflavin as a photosensitizer and potassium peroxydisulfate as a sacrificial oxidizing agent under visible light irradiation (Scheme 32). As a photocatalyst, riboflavin is an inexpensive natural reagent that shows important advances over transition-metal catalysis. In addition, the present method accepted a wide scope of functional groups and afforded the desired productions. This environmentally friendly procedure could be beneficial for pharmaceutical uses.

#### 2.2.2. Cyclization of CO_2_ as Raw Materials

**Scheme 33:**
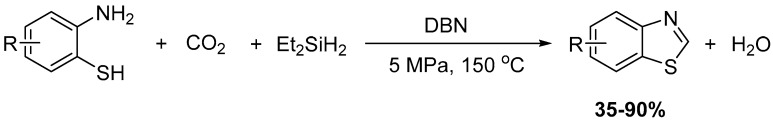
Cyclization of 2-aminothiophenols, CO_2_, and diethylsilane at 5 MPa.

Our group has reported a new route to synthesize benzothiazoles via cyclization of 2-aminothiophenols with CO_2_ in the presence of diethylsilane catalyzed by 1,5-diazabicyclo[4.3.0]non-5-ene (DBN) at 5 MPa, and various benzothiazoles were achieved in good yields (Scheme 33) [74]. The mechanism research shows that hydrosilane played an important role in the formation of benzothiazoles and suppressed the production of benzothiazolones as the byproducts. This study provides an environmentally benign approach for the synthesis of benzothiazoles.

**Scheme 34:**
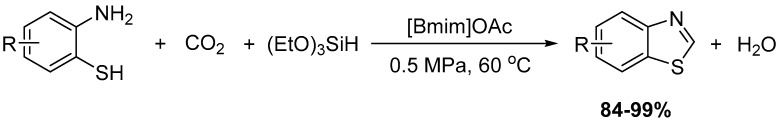
Cyclization of 2-aminobenzenethiols with CO_2_ and hydrosilane at 0.5 MPa.

In addition, the cyclization of 2-aminobenzenethiol compounds with CO_2_ and hydrosilane to produce a series of benzothiazoles was discovered by our group under mild conditions using acetate-based ionic liquid as a catalyst in high yields (Scheme 34) [75]. We have also investigated this reaction under different conditions with regard to various catalysts, temperature, and reaction pressure. Here, the best results were obtained by carrying out the reaction with 1-butyl-3-methylimidazolium acetate ([Bmim][OAc]) at 60 °C and 0.5 Mpa. Moreover, the reusability of [Bmim][OAc] was tested as well, and the yield of benzothiazole almost stayed unchanged as the IL was reused five times. Notably, this is the first protocol for the synthesis of benzothiazoles using CO_2_ as a raw material under metal-free and mild conditions.

**Scheme 35:**
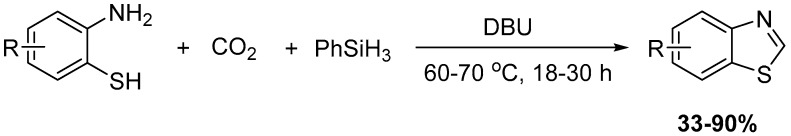
Cyclization of 2-aminobenzenethiols and CO_2_ at 0.1 MPa.

Chun and co-wokers [76] have reported that the synthesis of benzothiazoles from 2-aminobenzenethiols and carbon dioxide in the presence of 1,8-diazabicyclo[5.4.0]undec-7-ene (DBU) at 1 atm of CO_2_ and 60–70 °C (Scheme 35). The reaction had a broad substrate scope and functional group tolerance. Moreover, the precatalyst salt could be recovered and reused several times without any loss of activity.

## 3. Prospect for the Further Synthesis of Benzothiazoles

Even though the research on green synthesis methods for benzothiazoles has made many advances, the development of mild reaction conditions and inexpensive reaction systems is still a challenging problem. Meanwhile, the development of reaction systems with multiple green reaction conditions still needs to be explored by researchers. In the future, we will focus on designing and developing efficient, environmentally friendly, and economical reaction systems to make the reaction conditions of existing reaction pathways greener and the reaction process simpler, so as to realize the industrialized preparation of benzothiazole compounds and contribute to the development of green chemistry.

## 4. Conclusions

In conclusion, this review has summarized recent advances in the synthesis of benzothiazoles compounds related to green chemistry. In the past few decades, benzothiazoles have played a more and more important role in biochemistry and medicinal chemistry. As mentioned in this article, the condensation of 2-aminobenzenethiol with aldehydes/ketones/acids/acyl chlorides and the cyclization of thiamide or CO_2_ as raw materials have aroused more interest. Here, we have strived to compile most of these methods recently reported to support the development of various synthetic benzothiazole derivatives involving green chemistry. This work is of great significance for the development of synthetic pathways for benzothiazoles.

## References

[1] Carroll A.R., Scheuer P.J. (1990). pentacyclic alkaloids from a tunicate and its prosobranch mollusk predator Chelynotus semperi. J. Org. Chem..

[2] Gunawardana G.P., Kohmoto S., Gunasekera S.P., McConnel O.J., Koehn F.E. (1988). Dercitine, a new biologically active acridine alkaloid from a deep water marine sponge, Dercitus sp.. J. Am. Chem..

[3] Noel S., Cadet S., Gras E., Hureau C. (2013). The benzazole scaffold: A SWAT to combat Alzheimer’s disease. Chem. Soc. Rev..

[4] Prajapati N.P., Vekariya R.H., Borad M.A., Patel H.D. (2014). Recent advances in the synthesis of 2-substituted benzothiazoles: A review. RSC Adv..

[5] Kok S.H.L., Gambari R., Chui C.H., Yuen M.C.W., Lin E., Wong R.S.M., Lau F.Y., Cheng G.Y.M., Lam W.S., Chan S.H. (2008). Synthesis and anti-cancer activity of benzothiazole containing phthalimide on human carcinoma cell lines. Bioorg. Med. Chem..

[6] Heo Y., Song Y.S., Kim B.T., Heo J.N. (2006). A highly regioselective synthesis of 2-aryl-6-chlorobenzothiazoles employing microwave-promoted Suzuki–Miyaura coupling reaction. Tetrahedron. Lett..

[7] Alaimo R.J., Pelosi S.S., Freedman R. (1978). Synthesis and Antibacterial Evaluation of 2-(Substituted Phenylureido)-4-thiocyanatobenzothiazoles. J. Pharm. Sci..

[8] Singh M., Singh S.K., Gangwar M., Nath G., Singh S.K. (2014). Design, synthesis and mode of action of some benzothiazole derivatives bearing an amide moiety as antibacterial agents. RSC Adv..

[9] Ćaleta I., Grdiša M., Mrvoš-Sermek D., Cetina M., Tralić-Kulenovićd V., Pavelićb K., Karminski-Zamola K. (2004). Synthesis, crystal structure and antiproliferative evaluation of some new substituted benzothiazoles and styrylbenzothiazoles. Il Farmaco..

[10] Das J., Moquin R.V., Lin J., Liu C., Doweyko A.M., Defex H.F., Fang Q., Pang S., Pitt S., Shen D.R. (2003). Discovery of 2-amino-heteroaryl-benzothiazole-6-anilides as potent p56lck inhibitors. Bioorg. Med. Chem..

[11] Su X., Vicker N., Ganeshapillai D., Smith A., Purohit A., Reed M.J., Potter B.V.L. (2006). Benzothiazole derivatives as novel inhibitors of human 11β-hydroxysteroid dehydrogenase type 1. Mol. Cell. Endocrinol..

[12] Sreenivasa G.M., Jayachandran E., Shivakumar B., Jayaraj K.K., Vijay K.M.M.J. (2009). Synthesis of bioactive molecule fluoro benzothiazole comprising potent heterocyclic moieties for anthelmintic activity. Arch. Pharm. Sci. Res..

[13] Hutchinson I., Jennings S.A., Vishnuvajjala B.R., Westwell A.D., Stevens M.F.G. (2002). Antitumor benzothiazoles. 16. Synthesis and pharmaceutical properties of antitumor 2-(4-aminophenyl) benzothiazole amino acid prodrugs. J. Med. Chem..

[14] Bradshaw T.D., Westwell A.D. (2004). The development of the antitumour benzothiazole prodrug, Phortress, as a clinical candidate. Curr. Med. Chem..

[15] Yoshida M., Hayakawa I., Hayashi N., Agatsuma T., Oda Y., Tanzawa F., Iwasaki S., Koyama K., Furukawa H., Kurakata S. (2005). Synthesis and biological evaluation of benzothiazole derivatives as potent antitumor agents. Bioorg. Med. Chem. Lett..

[16] Vicini P., Geronikaki A., Incerti M., Busonera B., Poni G., Cabras C.A., Colla P.L. (2003). Synthesis and biological evaluation of benzo [d] isothiazole, benzothiazole and thiazole Schiff bases. Bioorg. Med. Chem,..

[17] Nagarajan S.R., Crescenzo G.A., Getman D.P., Lu H.F., Sikorski J.A., Walker J.L., Mcdonald J.J., Houseman K.A., Kocan G.P., Kishore N. (2003). Discovery of novel benzothiazole sulfonamides as potent inhibitors of HIV-1 protease. Bioorg. Med. Chem..

[18] Cressier D., Prouillac C., Hernandez P., Amourette C., Diserbo M., Lion C., Rima G. (2009). Synthesis, antioxidant properties and radioprotective effects of new benzothiazoles and thiadiazoles. Bioorg. Med. Chem..

[19] Dogruer D.S., Ünlü S., Şahin M.F., Yqilada E. (1998). Anti-nociceptive and anti-inflammatory activity of some (2-benzoxazolone-3-yl and 2-benzothiazolone-3-yl) acetic acid derivatives. Il Farmaco..

[20] El-Sherbeny M.A. (2000). Synthesis of certain pyrimido [2, 1-b] benzo-thiazole and benzothiazolo [2,3-b] quinazoline derivatives for in vitro antitumor and antiviral activities. Arzneimittelforschung..

[21] Jimonet P., Audiau F., Barreau M., Blanchard J.C., Boireau A., Bour Y., Coleno M.A., Doble A., Doerflinger G., Huu C.D. (1999). Riluzole series. Synthesis and in vivo “antiglutamate” activity of 6-substituted-2-benzothiazolamines and 3-substituted-2-imino-benzothiazolines. J. Med. Chem..

[22] Siddiqui N., Rana A., Khan S.A., Haque S.E., Alam M.S., Ahsan W., Ahmed S. (2009). Anticonvulsant and Toxicity Evaluation of Newly Synthesized 1-[2-(3, 4-disubstituted phenyl)-3-chloro-4-oxoazetidin-1-yl]-3-(6-substituted-1, 3-benzothiazol-2-yl) ureas. Acta Chim. Slov..

[23] Rajeeva B., Srinivasulu N., Shantakumar S.M. (2009). Synthesis and Antimicrobial Activity ofSome New 2-Substituted Benzothiazole Derivatives. J. Chem..

[24] Danzeisen R., Schwalenstoecker B., Gillardon F., Buerger E., Krzykalla V., Klinder K., Schild L., Hengerer B., Ludolph A.C., Dorner-Ciossek C. (2006). Targeted antioxidative and neuroprotective properties of the dopamine agonist pramipexole and its nondopaminergic enantiomer SND919CL2x [(+) 2-amino-4, 5, 6, 7-tetrahydro-6-Lpropylamino-benzathiazole dihydrochloride]. J. Pharmacol. Exp. Ther..

[25] Mylari B.L., Larson E.R., Beyer T.A., Zembrowski W.J., Aldinger C.E., Dee M.F., Siegel T.W., Singleton D.H. (1991). Novel, potent aldose reductase inhibitors: 3,4-dihydro-4-oxo-3-[[5-(trifluoromethyl)-2-benzothiazolyl] methyl]-1-phthalazineacetic acid (zopolrestat) and congeners. J. Med. Chem..

[26] Bahrami K., Khodaei M.M., Naali F. (2008). Mild and Highly Efficient Method for the Synthesis of 2-Arylbenzimidazoles and 2-Arylbenzothiazoles. J. Org. Chem..

[27] Panda S.S., Ibrahim M.A., Oliferenko A.A., Asiri A.M., Katritzky A.R. (2013). Catalyst-free facile synthesis of 2-substituted benzothiazoles. Green Chem..

[28] Riadi Y., Mamouni R., Azzalou R., Haddadc M.E., Guillaumetd S.R.G., Lazara S. (2011). An efficient and reusable heterogeneous catalyst animal bone meal for facile synthesis of benzimidazoles, benzoxazoles, and benzothiazoles. Tetrahedron. Lett..

[29] Sun Y., Jiang H., Wu W., Zeng W., Wu X. (2013). Copper-catalyzed synthesis of substituted benzothiazoles via condensation of 2-aminobenzenethiols with nitriles. Org. Lett..

[30] Xiao R., Hao W., Ai J., Cai M.Z. (2012). A practical synthesis of 2-aminobenzothiazoles via the tandem reactions of 2-haloanilines with isothiocyanates catalyzed by immobilization of copper in MCM-41. J. Org. Chem..

[31] Ma D., Lu X., Shi L., Zhang H., Jiang Y., Liu X. (2011). Domino Condensation/S-Arylation/Heterocyclization Reactions: Copper-Catalyzed Three-Component Synthesis of 2-N-Substituted Benzothiazoles. Angew. Chem. Int. Ed..

[32] Deng H., Li Z., Ke F., Zhou X. (2012). Cu-Catalyzed Three-Component Synthesis of Substituted Benzothiazoles in Water. Chem. Eur. J..

[33] Shi L., Liu X., Zhang H., Jiang Y., Ma D. (2011). Synthesis of 2-Thio-Substituted Benzothiazoles via a Domino Condensation/S-Arylation/Heterocyclization Process. J. Org. Chem..

[34] Ding Q., Huang X.G., Wu J. (2009). Facile Synthesis of Benzothiazoles via Cascade Reactions of 2-Iodoanilines, Acid Chlorides and Lawesson’s Reagent. J. Comb. Chem..

[35] Joyce L.L., Evindar G., Batey R.A. (2004). Copper- and palladium-catalyzed intramolecular C-S bond formation: A convenient synthesis of 2-aminobenzothiazoles. Chem. Commun..

[36] Cheng Q., Peng W., Fan P. (2014). Room-Temperature Ligand-Free Pd/C-Catalyzed C-S Bond Formation: Synthesis of 2-Substituted Benzothiazoles. J. Org. Chem..

[37] Sahoo S.K., Banerjee A., Chakraborty S., Patel B.K. (2012). Regioselective intramolecular arylthiolations by ligand free cu and pd catalyzed reaction. ACS Catal..

[38] Qi Y., Ma J., Chen X., Xiu F.R., Chen Y., Lu Y. (2020). Practical aptamer-based assay of heavy metal mercury ion in contaminated environmental samples: Convenience and sensitivity. Anal. Bioanal. Chem..

[39] Qi Y., Chen Y., Xiu F.R., Hou J. (2020). An aptamer-based colorimetric sensing of acetamiprid in environmental samples: Convenience, sensitivity and practicability. Sens. Actuators B Chem..

[40] Xiu F.R., Wang Y., Yu X., Li Y., Lu Y., Zhou K., He J., Song Z., Gao X. (2020). A novel safety treatment strategy of DEHP-rich flexible polyvinyl chloride waste through low-temperature critical aqueous ammonia treatment. Sci. Total Environ..

[41] Xiu F.R., Lu Y., Qi Y. (2020). DEHP degradation and dechlorination of polyvinyl chloride waste in subcritical water with alkali and ethanol: A comparative study. Chemosphere..

[42] Chen Y., Gao X., Liu X., Ji G., Fu L., Yang Y., Yu Q., Zhang W., Xue X. (2020). Water collection from air by ionic liquids for efficient visible-light-driven hydrogen evolution by metal-free conjugated polymer photocatalysts. Renew. Energ..

[43] Batista R.M., Costa S.P., Raposo M.M.M. (2004). Synthesis of new fluorescent 2-(2′, 2″-bithienyl)-1, 3-benzothiazoles. Tetrahedron Lett..

[44] Guo H.Y., Li J.C., Shang Y.L. (2009). A simple and efficient synthesis of 2-substituted benzothiazoles catalyzed by H_2_O_2_/HCl. Chin. Chem. Lett..

[45] Kumar A., Maurya R.A., Ahmad P. (2009). Diversity oriented synthesis of benzimidazole and benzoxa/(thia) zole libraries through polymer-supported hypervalent iodine reagent. J. Combin. Chem..

[46] Maleki B., Salehabadi H. (2010). Ammonium chloride; as a mild and efficient catalyst for the synthesis of some 2-arylbenzothiazoles and bisbenzothiazole derivatives. Eur. J. Chem..

[47] Praveen C., Nandakumar A., Dheenkumar P., Muralidharan D., Perumal P. (2012). Microwave-assisted one-pot synthesis of benzothiazole and benzoxazole libraries as analgesic agents. J. Chem. Sci..

[48] Ye L., Chen J., Mao P., Mao Z., Zhang X., Yan M. (2017). Visible-light-promoted synthesis of benzothiazoles from 2-aminothiophenols and aldehydes. Tetrahedron Lett..

[49] Maphupha M., Juma W.P., Koning C.B., Brady D. (2018). A modern and practical laccase-catalysed route suitable for the synthesis of 2-arylbenzimidazoles and 2-arylbenzothiazoles. RSC. Adv..

[50] Merroun Y., Chehab S., Ghailane T., Akhazzane M., Souizi1 A., Ghailane R. (2019). Preparation of tin-modified mono-ammonium phosphate fertilizer and its application as heterogeneous catalyst in the benzimidazoles and benzothiazoles synthesis. Reac Kine. Mech Cat..

[51] Bhat R., Karhale S., Arde S., Helavi V. (2019). Acacia concinna pod catalyzed synthesis of 2-arylbenzothia/(oxa) zole derivatives. Iran. J. Catal..

[52] Elderfield R.C., McClenachan E.C. (1960). Pyrolysis of the Products of the Reaction of o-Aminobenzenethiols with Ketones1. J. Am. Chem. Soc..

[53] Liao Y., Qi H., Chen S., Jiang P., Zhou W., Deng G.P. (2012). Efficient 2-Aryl Benzothiazole Formation from Aryl Ketones and 2-Aminobenzenethiols under Metal-Free Conditions. Org. Lett..

[54] Mayo M.S., Yu X., Zhou X., Feng X., Yamamoto Y., Bao M. (2014). Convenient synthesis of benzothiazoles and benzimidazoles through Brønsted acid catalyzed cyclization of 2-amino thiophenols/anilines with β-diketones. Org. Lett..

[55] Loukrakpam D.C., Phukan P. (2019). TsNBr_2_ Mediated Synthesis of 2-Acylbenzothiazoles and Quinoxalines from Aryl Methyl Ketones under Metal Free Condition. Chemistry Select..

[56] Gupta S.D., Singh H.P., Moorthy N. (2007). Iodine-Catalyzed, One-Pot, Solid-Phase Synthesis of Benzothiazole Derivatives. Synth Commun..

[57] Rauf A., Gangal S., Sharma S. (2008). Solvent-free synthesis of 2-alkyl and 2-alkenylbenzothiazoles from fatty acids under microwave irradiation. Indian J. Chem..

[58] Sharghi H., Asemani O. (2009). Methanesulfonic acid/SiO_2_ as an efficient combination for the synthesis of 2-substituted aromatic and aliphatic benzothiazoles from carboxylic acids. Synth Commun..

[59] Coelho F.L., Campo L.F. (2017). Synthesis of 2-arylbenzothiazoles via direct condensation between in situ generated 2-aminothiophenol from disulfide cleavage and carboxylic acids. Tetrahedron Lett..

[60] Nadaf R.N., Siddiqui S.A., Daniel T., Lahoti R.J., Srinivasan K.V. (2004). Room temperature ionic liquid promoted regioselective synthesis of 2-aryl benzimidazoles, benzoxazoles and benzthiazoles under ambient conditions. J. Mol. Catal. A: Chem..

[61] Wu C., Wei J., Gao K., Wang Y. (2007). Dibenzothiazoles as novel amyloid-imaging agents. Bioorg. Med. Chem..

[62] Racané L., Kralj M., Šuman L., Stojkovićc R., Tralić-Kulenovićb V., Karminski-Zamola G. (2010). Novel amidino substituted 2-phenylbenzothiazoles: Synthesis, antitumor evaluation in vitro and acute toxicity testing in vivo. Bioorg. Med. Chem..

[63] Kumar K.R., Satyanarayana P.V.V., Reddy B.S. (2012). NaHSO_4_-SiO_2_-Promoted Solvent-Free Synthesis of Benzoxazoles, Benzimidazoles, and Benzothiazole Derivatives. J. Chem..

[64] Dar A.A., Shadab M., Khan S., Ali N., Khan A.T. (2016). One-pot synthesis and evaluation of antileishmanial activities of functionalized S-alkyl/aryl benzothiazole-2-carbothioate scaffold. J. Org. Chem..

[65] Luo B., Li D., Zhang A.L., Gao J. (2018). Synthesis, antifungal activities and molecular docking studies of benzoxazole and benzothiazole derivatives. Molecules..

[66] Bose D.S., Idrees M. (2006). Hypervalent Iodine Mediated Intramolecular Cyclization of Thioformanilides:  Expeditious Approach to 2-Substituted Benzothiazoles. J. Org. Chem..

[67] Downer-Riley N.K., Jackson Y.A. (2008). Conversion of thiobenzamides to benzothiazoles via intramolecular cyclization of the aryl radical cation. Tetrahedron..

[68] Rey V., Soria-Castro S.M., Argueello J.E., Peñéñory A.B. (2009). Photochemical cyclization of thioformanilides by chloranil: An approach to 2-substituted benzothiazoles. Tetrahedron Lett..

[69] Feng E., Huang H., Zhou Y., Ye D., Jiang H., Liu H. (2010). Metal-Free Synthesis of 2-Substituted (N, O, C) Benzothiazoles via an Intramolecular C− S Bond Formation. J. Combin Chem..

[70] Cheng Y., Yang J., Qu Y., Li P. (2012). Aerobic visible-light photoredox radical C–H functionalization: Catalytic synthesis of 2-substituted benzothiazoles. Org. Lett..

[71] Folgueiras-Amador A.A., Qian X.Y., Xu H.C., Wirth T. (2018). Catalyst-and supporting-electrolyte-free electrosynthesis of benzothiazoles and thiazolopyridines in continuous flow. Chem. Eur. J..

[72] Xu Z.M., Li H.X., Young D.J., Zhu D.L., Li H.Y., Lang J.P. (2018). Exogenous photosensitizer-, metal-, and base-free visible-light-promoted C–H thiolation via reverse hydrogen atom transfer. Org. Lett..

[73] Bouchet L.M., Heredia A.A., Arguello J.E., Schmidit L.C. (2019). Riboflavin as Photoredox Catalyst in the Cyclization of Thiobenzanilides: Synthesis of 2-Substituted Benzothiazoles. Org. Lett..

[74] Gao X., Yu B., Zhao Y., Hao L., Liu C. (2014). Hydrosilane-promoted cyclization of 2-aminothiophenols by CO_2_ to benzothiazoles. RSC Adv..

[75] Gao X., Yu B., Yang Z., Zhao Y., Zhang H., Hao L., Han B., Liu Z. (2015). Ionic liquid-catalyzed C–S bond construction using CO_2_ as a C1 building block under mild conditions: A metal-free route to synthesis of benzothiazoles. ACS Catal..

[76] Chun S., Yang S., Chung Y.K. (2017). Synthesis of benzothiazoles from 2-aminobenzenethiols in the presence of a reusable polythiazolium precatalyst under atmospheric pressure of carbon dioxide. Tetrahedron..

